# New approach methodologies in neurotherapeutics development

**DOI:** 10.1016/j.neurot.2026.e01056

**Published:** 2026-09-17

**Authors:** Amir P. Tamiz, Szczepan W. Baran, Thomas Hartung, Wendy W. Wu

**Affiliations:** a Division of Translational Research, National Institute of Neurological Disorders and Stroke, NIH, 6001 Executive Blvd., Rockville, MD, 20852, USA; b Szczepan Baran, Chief Scientific Officer, Instem, 800 Boylston Street, Suite 1450, Boston, MA, 02199, USA; c Doerenkamp-Zbinden Professor and Chair for Evidence-based Toxicology, Johns Hopkins University, Baltimore, MD, 21205, USA; d Division of Applied Regulatory Science, Office of Clinical Pharmacology, Office of Translational Sciences, Center for Drug Evaluation and Research, US Food and Drug Administration, Silver Spring, MD, USA

**Keywords:** New approach methodologies, Neurotherapeutics discovery, Neurotherapeutics development, Regulatory, Reproducibility and validation

## Abstract

New Approach Methodologies (NAMs) offer substantial opportunities to transform neurotherapeutics discovery, optimization, and development, reducing the timeline to translate central nervous system (CNS) innovations to patients. Strategically deployed, NAMs enhance the predictive value of preclinical studies for human outcomes while reducing reliance on animal models. Current scientific community interest focuses particularly on human-based and -derived systems, *in silico* and AI-driven models, and advanced microphysiological platforms, reflecting a shift toward human-centric drug development paradigms. Despite this momentum, significant challenges remain, including NAM reproducibility and validation, the establishment of standardized performance criteria, data sharing, and the evolution of regulatory frameworks needed to enable consistent adoption across the neurotherapeutics continuum. Nevertheless, there is a rich history of developing and adopting methodologies, particularly for improving the prediction of neurotherapeutic safety profiles while reducing animal use, and for advancing understanding of drug delivery across the blood–brain barrier, and the assessment of adverse neurological effects. These advances have begun to influence regulatory decision-making and are increasingly reflected in guidance and review practices. Furthermore, NAMs are showing concrete impact in neurological disorders, including Epilepsy, Amyotrophic lateral sclerosis (ALS), Alzheimer’s disease, and Parkinson’s disease, where human-relevant models and computational approaches support more precise characterization of disease mechanisms and therapeutic responses. In this paper, we examine the current and emerging roles of NAMs in neurotherapeutics development from government, academia, and industry perspectives, highlight key opportunities and limitations, and discuss the scientific, technical, and regulatory steps required to fully realize their potential in accelerating safe and effective CNS therapies.

Neurotherapeutics development has historically been challenging, primarily because of our limited understanding of the brain, including its complex architecture and connectivity and how various conditions affect its function. Recent advances in molecular biology and imaging technologies have significantly improved our understanding of brain physiology and function. Together, these innovations have also created opportunities for scientists to interrogate diseases and conditions directly in human-based and -derived systems that more accurately reflect human biology and disease.

The current state of brain science, together with emerging drug modalities such as amino-acid- and nucleic-acid-based products, will likely shape the near future of delivering meaningful symptomatic and disease-modifying treatments for patients. Key questions now are: 1) how best to refine experimental models to more accurately predict utility and proof of concept for personalized interventions, 2) how best to deliver them to the right target, 3) and how best to assess their safety while simultaneously reducing our traditional reliance on animal models, which have long exhibited predictive and ethical limitations in modeling human conditions.

New approach methodologies (NAMs) have already been successfully applied in later preclinical stages of therapeutics development, particularly in toxicology and ADME (Absorption, Distribution, Metabolism, and Excretion) studies, to evaluate the suitability of neurotherapeutic candidates [1,2]. NAMs utilized across the translational spectrum can improve overall druggability predictions from initial therapeutic selection through optimization and final candidate selection and can strengthen measures of drug target potency and selectivity while helping interrogate large drug libraries. However, more sophisticated and scalable NAMs are still needed to match the complexity, speed, and cost of neurotherapeutics development within the regulatory framework, so that therapies can advance to patients more efficiently.

Applied earlier in discovery, NAMs can similarly de-risk the progression of projects into clinical studies and increase the likelihood that candidate drugs will have the properties and durability needed to benefit patients. Realizing this potential across the pipeline from early discovery through late-stage development will require addressing persistent challenges such as transparency, data sharing, standardized reporting, and context of use, alongside rigorous experimental validation to support broad adoption in both academia and industry. This moment presents an important opportunity for funders and regulators to provide the support needed for widespread implementation and sustained impact of NAMs in neurotherapeutics.

## NIH Perspective: Catalyzing Development and Use of NAMs to Advance Biomedical Research

Neurotherapeutics remains one of the most difficult domains in biomedical research, with high attrition rates and persistent gaps between preclinical findings and clinical outcomes. A major reason is that conventional animal models often incompletely capture human brain biology, disease heterogeneity, and treatment response. NAMs offer a more human-centered path by enabling mechanistic, scalable, and often patient-specific models of the nervous system [3,4]. In this context, the NIH Complement Animal Research In Experimentation (Complement-ARIE) Program and the NIH Office of Research Innovation, Validation, and Application (ORIVA) can serve as complementary engines for accelerating translation by establishing a strong development-to-deployment pathway for NAMs in neurotherapeutics.

The Complement-ARIE program is intended to catalyze upstream innovation by supporting multidisciplinary teams that integrate stem cell biology, neuroengineering, microphysiological systems, computational modeling, imaging, and data science to build fit-for-purpose platforms for central nervous system disorders [5]. In neurotherapeutics, these platforms include human induced pluripotent stem cell-derived neurons and glia, brain organoids and assembloids, blood-brain barrier and neurovascular unit models, and *in silico* tools capable of capturing disease mechanisms, target engagement, and neurotoxicity in human-relevant contexts [1,3,5,6]. Such models may especially be valuable for disorders with high translational attrition, including Alzheimer’s disease, Parkinson’s disease, amyotrophic lateral sclerosis, epilepsy, and rare neurogenetic conditions.

ORIVA aims to extend this impact by addressing barriers to NAM adoption. Innovative models will not influence drug development unless investigators, regulators, funders, and industry can determine that they are reproducible, reliable, and fit for a defined context of use. ORIVA can help establish consensus performance standards, benchmarking frameworks, shared datasets, and transparent validation pathways aligned with good *in vitro* method practices and regulatory needs [3,7,8]. For neuroscience, where outcomes depend on complex multicellular interactions and long disease trajectories, such infrastructure is essential.

**Fig. 1:**
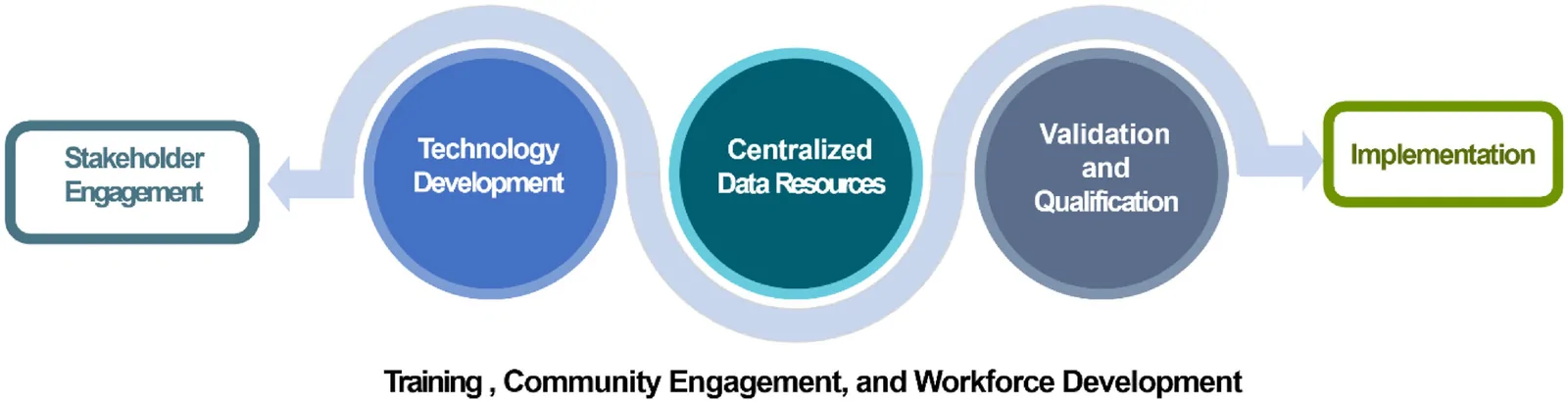
**Training, Community Engagement and Workforce Development.** Components for successful adoption and implementation of NAMs include technology development, centralized data resources, validation and qualification, along with key training and dissemination activities to provide a fluid conduit from engagement to implementation.

Together, Complement-ARIE and ORIVA can create a translation-ready ecosystem by linking innovation, validation, interoperability, and implementation (Fig. 1). Coordinated investments in data standards, FAIR data sharing, training, and public-private partnerships can help move NAMs from promising prototypes to decision-ready tools. Anchoring these platforms to patient-derived samples and clinical outcome data will further strengthen their human relevance. By advancing rigorous, human-centered evidence generation, Complement-ARIE and ORIVA can improve confidence in NAMs for neurotherapeutics development and ultimately support safer, more effective interventions for neurological disorders. This comprehensive ecosystem can minimize translational failures, reduce development costs, and deliver targeted neurotherapeutics to patients faster.

## Academic Perspective: From Brain Organoids to Organoid Intelligence

**Fig. 2:**
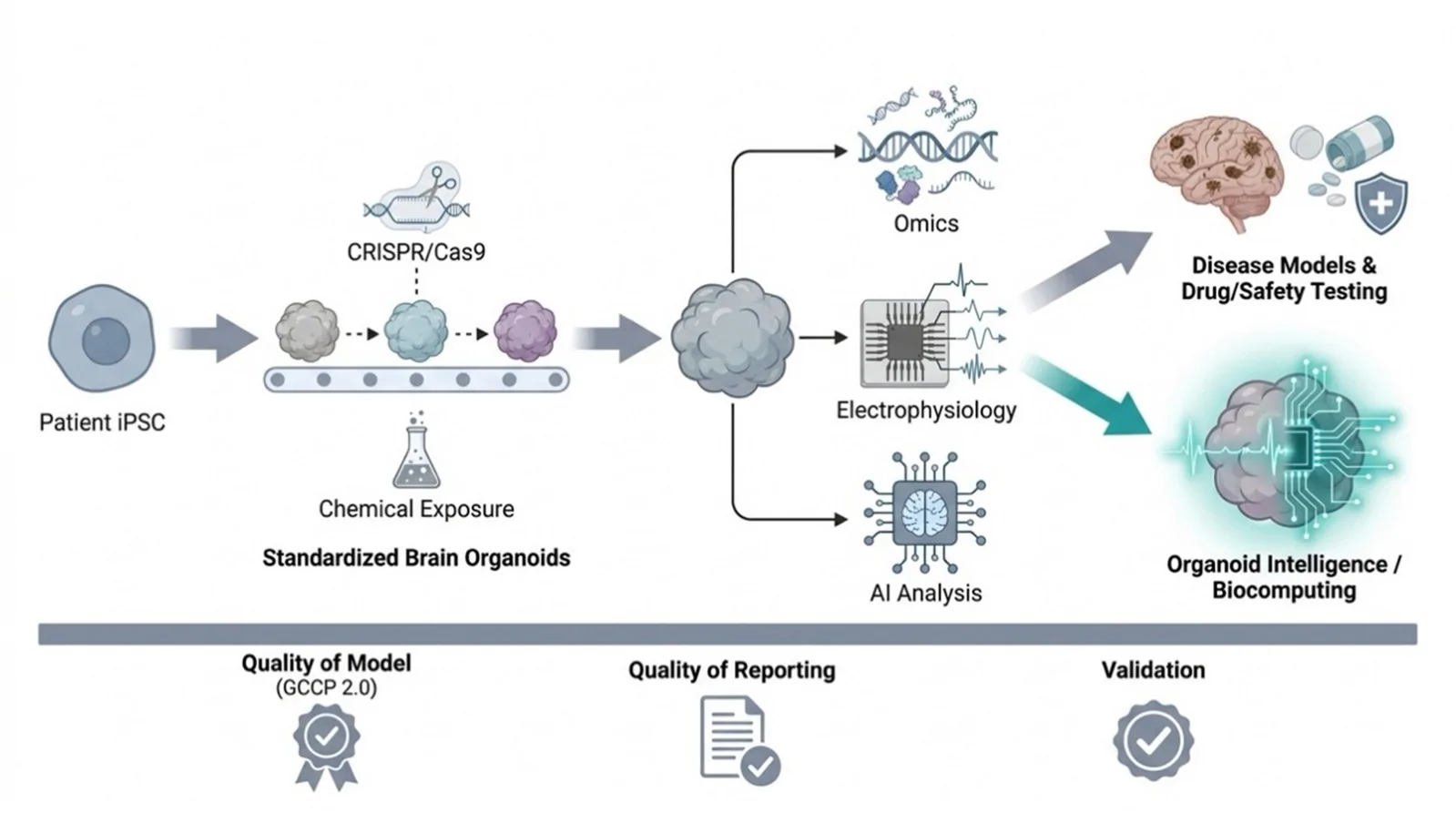
**The academic NAM pipeline for neurotherapeutics.** NAM stages of development guided across quality attributes: Model building (MPS and exposure), read-outs, micropathophysiological systems and OI as functional read-out, and Quality Assurance (QA) layer. This Figure was generated using publicly available version of ChatGPT and reviewed for accuracy and content by the authors.

Academia’s distinctive contribution to NAMs is to generate and de-risk disruptive technologies before regulators and industry can adopt them. Over the past decade, the academic research has arrived at convergence of human induced pluripotent stem cell (iPSC) biology, microphysiological systems (MPS), multi-omics, biosensors, and artificial intelligence (AI), moving human-relevant neuroscience from promise to a working toolkit (Fig. 2). A pivotal step was the demonstration that three-dimensional human brain MPS could be standardized and mass-produced from iPSCs, yielding reproducible neuronal–glial cultures with spontaneous electrophysiological activity suitable for disease modeling and neurotoxicity testing [9]. Notably, enabling long-term integration of microglia, the brain’s resident immune cells, into brain organoids added inflammatory pathomechanisms [10].

These models give experimental access to physiology that animal and conventional *in vitro* systems cannot capture. Because organoids carry the donor’s genetic background and can be edited with CRISPR/Cas9, they uniquely enable the study of gene–environment interactions. For example, recent studies have shown functional and molecular synergy between knockout of the autism-risk gene CHD8 and exposure to the pesticide chlorpyrifos in human brain organoids [11]. For neurotherapeutics, this opens patient- and disease-specific platforms for target identification, efficacy screening, and safety assessment.

The frontier is to harness the brain’s own computational capacity. Organoid Intelligence (OI) aims to realize learning and memory in brain MPS, both to study the biological basis of cognition and to build a new generation of functional, information-processing assays for drug and chemical testing [12]. Such models may, in time, complement silicon computing while serving as the most physiologically complete experimental systems for neurodevelopment and neurological disease. An ethical framework is necessary to support vital research in this promising field while addressing public perceptions.

The rate-limiting step for adoption is not capability but trust. Academia must therefore pair innovation with rigor across three axes: quality of the cell model (Good Cell and Tissue Culture Practice 2.0) [13], quality of reporting (good *in vitro* reporting standards), and quality of results (validation) [9] AI serves this agenda twice over: as a NAM, where machine learning on toxicological big data already rivals the reproducibility of animal tests [14], and, increasingly, as an agentic tool that audits compliance with reporting guidance. Standardization is how academic discovery becomes regulatory currency, and it is the bridge between the academic, governmental, and industrial perspectives represented.

## Industry perspective: from conflation to decisional integration

Much of the current debate treats NAMs as replacements for animal studies. This framing inherits a category error. NAMs matured inside chemical hazard assessment under the pressure of volume: thousands of untested compounds need to be triaged faster than animal studies allow, and high-throughput *in vitro* and *in silico* assays, validated for that screening job, solve it well [15]. Hazard still must be called correctly there, but a wrong call sends a compound up the testing tier, not into a patient. That is translation, not triage, and it is where late-stage attrition lands [16]. Notably, clinical failure rates are higher in the central nervous system than in any other therapeutic area, most often due to lack of efficacy, even when preclinical data looked promising [17]. A method proven at sorting thousands is not thereby proven at predicting one. Same toolbox, different bench.

**Fig. 3:**
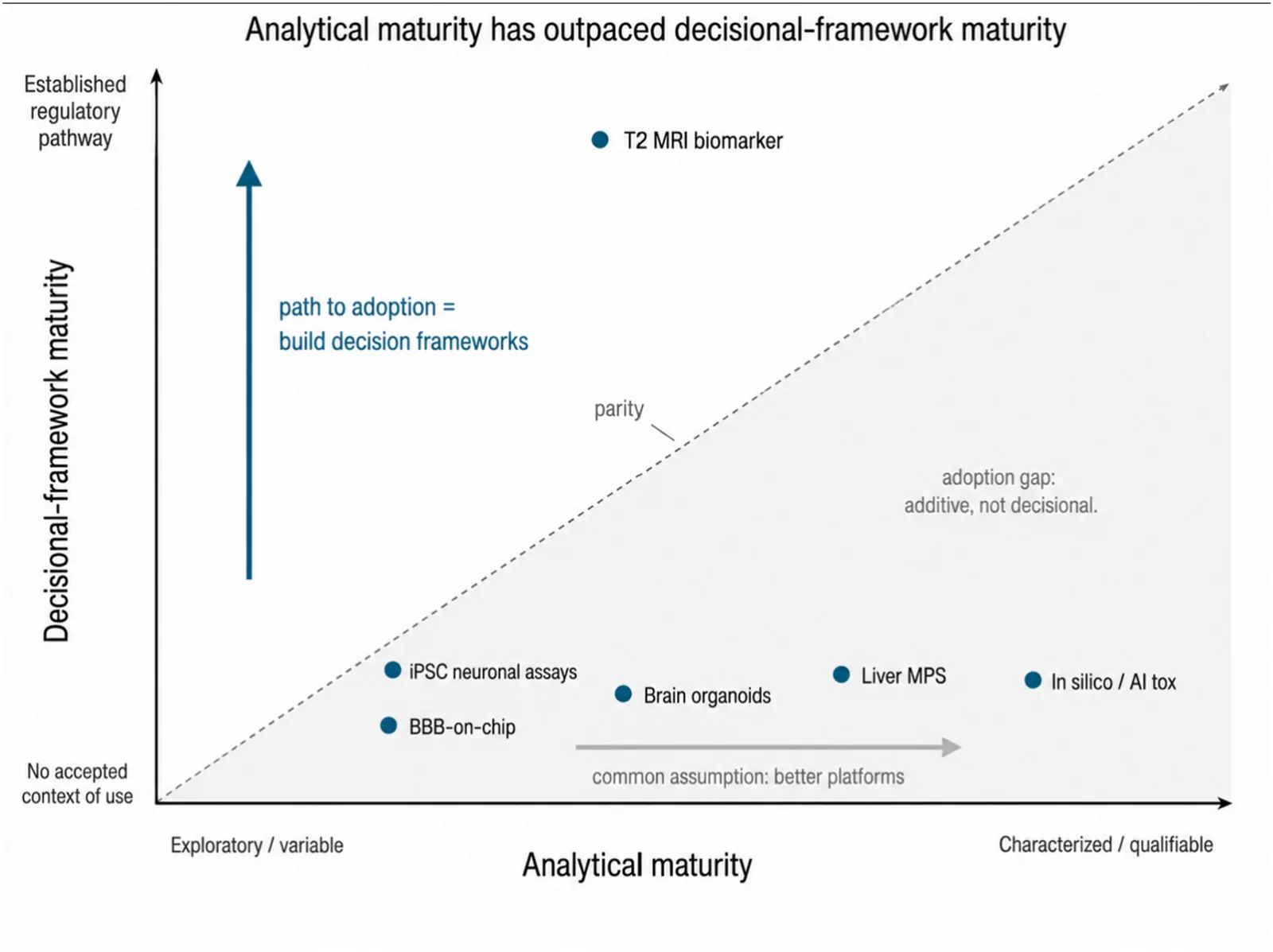
**Analytical maturity has outpaced decisional-framework maturity for neuro NAMs.** Positioning of NAMs on two independent axes: analytical maturity (reproducibility and qualifiability of the measurement itself) and decisional-framework maturity (whether an accepted context of use, reference ranges, and a regulatory pathway exist for acting on the result). Most methods fall below the diagonal, indicating that technical development has run ahead of the frameworks needed to make results decisional; the imaging biomarker entering formal qualification is the exception that marks the vertical path. The liver microphysiological node sits low on decisional maturity because the same endpoints are reported in incompatible units across platforms, leaving regulators without reference ranges to interpret them. This Figure was generated using publicly available version of ChatGPT and reviewed for accuracy and content by the authors.

The barrier industry typically hits in practice is not whether the science is good enough, or whether the law still requires animal studies [18]. Regulatory willingness now runs ahead of practitioner readiness [19]. Most NAMs remain additive: they generate a signal without a framework that tells us what decision it supports. A liver microphysiological system can report hepatocellular injury, but sponsors cannot compare that readout across contract research organizations because platforms report the same endpoints in incompatible units, and regulators lack reference ranges to interpret them [20]. The iPSC-derived neuronal assays are no more comparable, varying in cell type and proportion between laboratories (Fig. 3). The harder problem is not a better assay. It is the context of use, the reference data, and the qualification path that make a result decisional rather than merely interesting [21,22].

For microphysiological systems, industry often has workable reporting conventions; for AI and machine learning outputs are not common, and a model trained on a million points is not obviously an N of one or an N of a million. What a model must demonstrate in statistical performance, reproducibility, and explainability should scale with how much of the decision it carries [23]. Incentives stall it next. Regulators ask for the translation data that would build their confidence, but a sponsor who submits an ambiguous signal absorbs the delay while the field takes the benefit. Until that asymmetry is addressed, the evidence that would establish context of use stays inside companies. Precompetitive work such as the IQ MPS Affiliate is how both get fixed [24].

Adoption is accelerated by developing effective methods that fully meet the 3Rs (Reduce, Replace, Refine), rather than simply checking them off. Industry moves fastest when a method is grounded in relevant biology, mirrors human mechanisms, and predicts a patient outcome that informs a real decision because that is also where the strongest case for replacement gets made. A promising signal is a good start, but what earns adoption is something that de-risks a program, and that rigor is what gives a new method credibility with regulators, investors, and partners alike. The reduction in animal usage stems directly from the increased rigor of alternative methods. When these approaches demonstrate their capacity to reliably substitute for animal studies in specific decision-making processes, the need for animal involvement decreases not due to a diminished priority, but because responsible standards have been fulfilled. Earning that trust means industry will comply with the regulatory framework, because full acceptance, not partial acceptance, is what protects both good science and animal welfare. The real opportunity is integration: rather than crowning one method the winner, industry gets to build a future where validated methods work together, each used exactly where it meets the rigor needed to replace animal testing for good.

## FDA activities to facilitate NAMs utilization

FDA’s support for integrating NAMs into regulatory review has been building steadily, driven by a straightforward goal: bring safe and effective therapies to patients faster while reducing dependence on animal testing. The agency’s evolving framework reflects a practical orientation understanding where NAM data align with clinical outcomes and how that data can support sound regulatory decisions.

Several recent developments mark meaningful progress. In 2022, the FDA Modernization Act 2.0 officially replaced the phrase “preclinical tests (including tests on animals)” in the 1938 Federal Food, Drug, and Cosmetic Act (specifically 21 U.S.C. § 355(i)) with the broader term “nonclinical tests.” Building on that legislative change, FDA issued its April 2025 “Roadmap to Reducing Animal Studies in Preclinical Safety Studies,” signaling a commitment to transitioning away from animal test methods. Most recently, in March 2026, the Center for Drug Evaluation and Research (CDER) released draft guidance titled “General Considerations for the Use of New Approach Methodologies in Drug Development.” That guidance clarifies that NAMs constitute part of a Weight-of-Evidence (WoE) approach, that may be applied in a fit-for-purpose manner without requiring formal qualification programs, and outlines key considerations for improving the interpretability of NAM data. Taken together, these developments confirm that FDA already acknowledges NAMs as a legitimate, current component of the evidentiary framework supporting drug development.

Within CDER, several initiatives are ongoing: clarifying NAM submission pathways for sponsors, involving appropriate subject matter experts in reviewing materials, and furthering understanding of how NAMs are utilized in submissions. The CDER NAMs Coordinating Committee drives the agency’s NAMs agenda through cross-center coordination, strategic direction, and external partnerships with NIH and international regulators, covering policy development, research, and data sharing. The Committee also launched the pilot Integrated Review Team for NAMs, which brings together multidisciplinary subject matter experts to evaluate NAM submissions consistently and assess how the resulting data may support safety and toxicology evaluations.

An internal research effort is also underway to catalog neuro related NAMs submitted through regulatory applications. The goal is to better characterize their intended context of use, the review decisions they informed whether as supporting or pivotal evidence and the limitations identified during review. Findings from this effort are intended to be shared externally, with the aim of improving the applicability and interpretability of neural NAM data across safety and efficacy assessments.

## Key Takeaways

We stand at an inflection point in how the world develops treatments for the brain. New Approach Methodologies represent a fundamentally more human-centered way of understanding disease - one that promises to close the long-standing gap between laboratory discovery and real benefit to patients.

Across NIH, academia, industry, and the FDA, a shared vision is taking shape: a future where brain organoids and human-derived models let us see disease mechanisms with unprecedented clarity; where AI and computational tools accelerate discovery; and where regulatory pathways evolve in step with the science, while advancing therapies to patients more efficiently. The FDA’s recent guidance, NIH’s investment in translation-ready infrastructure, and academia’s breakthroughs in NAMs all point in the same direction toward a system where reliable, human-relevant evidence can move confidently from bench to bedside.

The remaining challenges of standardization, data sharing, validation, and decisional frameworks are not obstacles so much as the final architecture still being built. Each sector is already contributing a piece: NIH is de-risking innovation, academia is proving what’s scientifically possible, industry is defining what makes evidence decision-ready, and regulators are opening the door wider than ever before.

If this momentum is sustained through coordinated investment and rigorous collaboration, the payoff is substantial: faster, safer, more precise therapies for devastating neurological conditions including Alzheimer’s, Parkinson’s, ALS, and epilepsy delivered to patients who have waited far too long. The tools to get there are rapidly evolving. What’s needed now is the shared vision to bring them fully to life.

## Author Contribution

Amir P. Tamiz, is responsible for all sections of this article, particularly the NIH perspective section.

Szczepan Baran, is responsible for drafting the industry perspective section, introductions, and overall takeaway conclusion.

Thomas Hartung, is responsible for drafting the academia perspective section, introductions, and overall takeaway conclusion.

Wendy W. Wu§, is responsible for drafting the FDA perspective section, introductions, and overall takeaway conclusion.

## Disclosures

S.W.B. is Chief Scientific Officer at Instem, a life sciences software and services provider spanning *in silico* prediction, nonclinical safety assessment, regulatory submission, and clinical trial analytics. He leads Instem’s scientific and AI strategy, including new approach methodologies (NAMs) and regulatory science. He is CEO and a founding board director of the Digital Preclinical Society, President of the 3Rs Collaborative, Industry Co-Chair of the FNIH/NIH Validation and Qualification Network, and principal at Baran Café, an independent consultancy. He has contributed to workshops of the IQ MPS Affiliate, publications from which are cited in this article. The views expressed are those of the author and do not necessarily represent those of these organizations.

## Declaration of AI and AI-asisted technologies in the manuscript preparation process

During the preparation of this work, the author(s) used a publicly available version of ChatGPT in order to help generate explanatory images in Figures 2 and 3. After using this tool/service, the author(s) reviewed and edited the content as needed and take(s) full responsibility for the content of the published article.

## Declaration of competing interest

The authors declare that they have no known competing financial interests or personal relationships that could have appeared to influence the work reported in this paper.

## References

[1] Imberechts D., Ny Annelii, Copmans D. (2025). Established and emerging new approach methodologies in neuroscience. Sec. Neuroscience methods and techniques. Front Neurosci.

[2] Wu X., Wu M., Zou J., Kleinstreuer N., Wu J. (2026). Reimagining human-centric drug development with new approach methodologies. Science.

[3] Low L.A., Mummery C., Berridge B.R., Austin C.P., Tagle D.A. (2021). Organs-on-chips: into the next decade. Nat Rev Drug Discov.

[4] Paşca S.P. (2022). Assembling human brain organoids. Science.

[5] Sunderic K., Wright A., Kleinstreuer N., Ledbetter V., Milora K., Happel C. (2025). Complement-ARIE: catalyzing the development and adoption of new approach methodologies. NAMTA J.

[6] Kim J., Koo B.-K., Knoblich J.A. (2020). Human organoids: model systems for human biology and medicine. Nat Rev Mol Cell Biol.

[7] OECD (2018).

[8] National Institutes of Health Office of research innovation, validation, and application (ORIVA). NIH. https://dpcpsi.nih.gov/oriva.

[9] Pamies D., Barreras P., Block K., Makri G., Kumar A., Wiersma D. (2017). A human brain microphysiological system derived from induced pluripotent stem cells to study neurological diseases and toxicity. ALTEX.

[10] Rittenhouse A., Krall C., Plotkin J., Alam El Din D.M., Kincaid B., Laird J. (2025). Microglia-containing neural organoids as brain microphysiological systems for long-term culture. Front Cell Neurosci.

[11] Modafferi S., Zhong X., Kleensang A., Murata Y., Fagiani F., Pamies D. (2021). Gene-environment interactions in developmental neurotoxicity: a case study of synergy between chlorpyrifos and CHD8 knockout in human BrainSpheres. Environ Health Perspect.

[12] Smirnova L., Caffo B.S., Gracias D.H., Huang Q., Morales Pantoja I.E., Tang B. (2023). Organoid intelligence (OI): the new frontier in biocomputing and intelligence-in-a-dish. Front Sci.

[13] Pamies D., Leist M., Coecke S., Bowe G., Allen D.G., Gstraunthaler G. (2022). Guidance document on good cell and tissue culture practice 2.0 (GCCP 2.0). ALTEX.

[14] Luechtefeld T., Marsh D., Rowlands C., Hartung T. (2018). Machine learning of toxicological big data enables read-across structure activity relationships (RASAR) outperforming animal test reproducibility. Toxicol Sci.

[15] Dix D.J., Houck K.A., Martin M.T., Richard A.M., Setzer R.W., Kavlock R.J. (2007). The ToxCast program for prioritizing toxicity testing of environmental chemicals. Toxicol Sci.

[16] Waring M.J., Arrowsmith J., Leach A.R., Leeson P.D., Mandrell S., Owen R.M. (2015). An analysis of the attrition of drug candidates from four major pharmaceutical companies. Nat Rev Drug Discov.

[17] Gribkoff V.K., Kaczmarek L.K. (2017). The need for new approaches in CNS drug discovery: why drugs have failed, and what can be done to improve outcomes. Neuropharmacology.

[18] (2022). FDA modernization Act 2.0, S.5002, 117th Cong.

[19] Ewart L., Roth A. (2021). Opportunities and challenges with microphysiological systems: a pharma end-user perspective. Nat Rev Drug Discov.

[20] Tomlinson L., Ramsden D., Leite S.B., Beken S., Bonzo J.A., Brown P. (2024). Considerations from an international regulatory and pharmaceutical industry (IQ MPS Affiliate) workshop on the standardization of complex *in vitro* models in drug development. Adv Biol.

[21] Parish S.T., Aschner M., Casey W., Corvaro M., Embry M.R., Fitzpatrick S. (2020). An evaluation framework for new approach methodologies (NAMs) for human health safety assessment. Regul Toxicol Pharmacol.

[22] van der Zalm A.J., Barroso J., Browne P., Casey W., Gordon J., Henry T.R. (2022). A framework for establishing scientific confidence in new approach methodologies. Arch Toxicol.

[23] Tong W., Baran S.W. (2024). 50 shades of AI in regulatory science. Drug Discov Today.

[24] Baran S.W., Brown P.C., Baudy A.R., Fitzpatrick S.C., Frantz C., Fullerton A. (2022). Perspectives on the evaluation and adoption of complex *in vitro* models in drug development: workshop with the FDA and the pharmaceutical industry (IQ MPS Affiliate). ALTEX.

